# Supplementary figures and images for: Analysis of Tumor-Infiltrating T-Cell Transcriptomes Reveal a Unique Genetic Signature across Different Types of Cancer
Source: Int J Mol Sci. 2022 Sep 21;23(19):11065. doi: 10.3390/ijms231911065 (PMC9569723; doi:10.3390/ijms231911065)

A

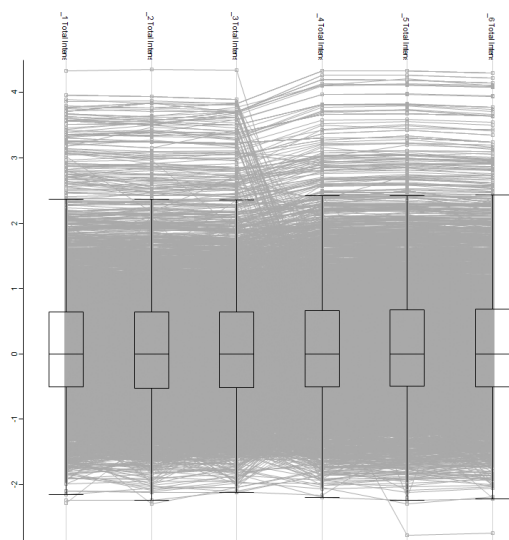

B

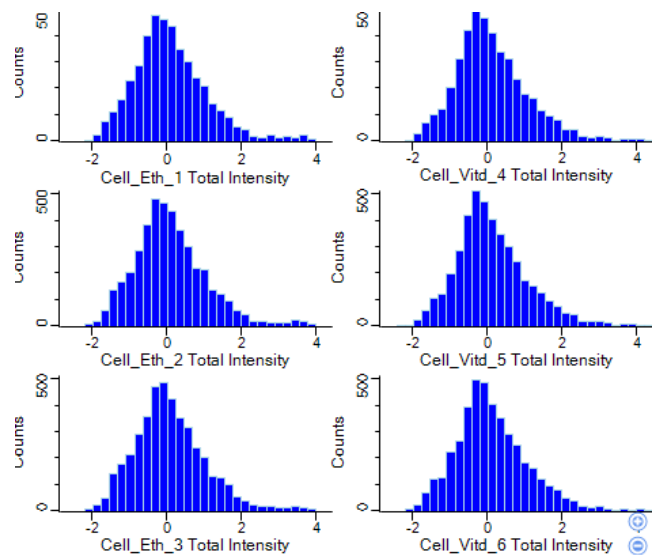

C

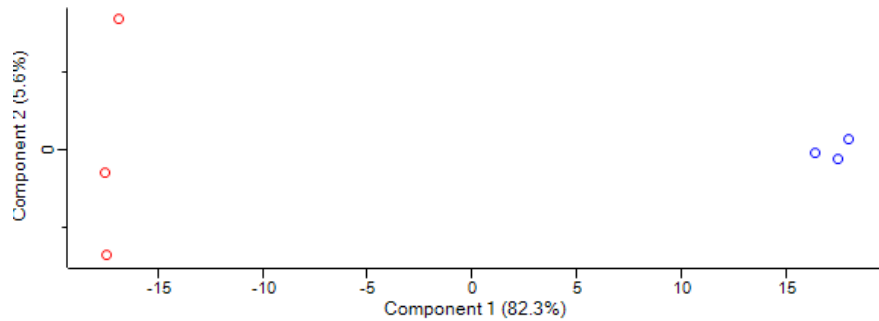

E

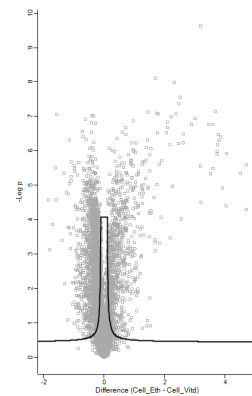

D

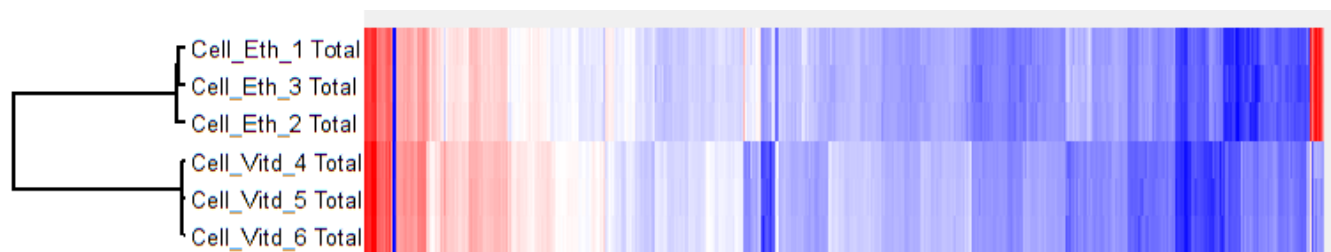

Supplement: Supplementary file 1 [file ijms-23-11065-s001.zip › Figure S1.pdf]
